# Effects of membrane potentials on the electroporation of giant unilamellar vesicles

**DOI:** 10.1371/journal.pone.0291496

**Published:** 2023-09-12

**Authors:** Md. Abdul Wadud, Mohammad Abu Sayem Karal, Md. Moniruzzaman, Md. Mamun Or Rashid

**Affiliations:** 1 Department of Physics, Bangladesh University of Engineering and Technology, Dhaka, Bangladesh; 2 Department of Pharmacy, Noakhali Science and Technology University, Noakhali, Bangladesh; East China Normal University School of Life Sciences, CHINA

## Abstract

Living organisms maintain a resting membrane potential, which plays an important role in various biophysical and biological processes. In the context of medical applications, irreversible electroporation (IRE) is a non-thermal and minimally invasive technique that utilizes precisely controlled electric field pulses of micro- to millisecond durations to effectively ablate cancer and tumor cells. Previous studies on IRE-induced rupture of cell-mimetic giant unilamellar vesicles (GUVs) have primarily been conducted in the absence of membrane potentials. In this study, we investigated the electroporation of GUVs, including parameters such as the rate constant of rupture and the probability of rupture, in the presence of various negative membrane potentials. The membranes of GUVs were prepared using lipids and channel forming proteins. As the membrane potential increased from 0 to −90 mV, the rate constant of rupture showed a significant increase from (7.5 ± 1.6)×10^−3^ to (35.6 ± 5.5)×10^−3^ s^-1^. The corresponding probability of rupture also exhibited a notable increase from 0.40 ± 0.05 to 0.68 ± 0.05. To estimate the pore edge tension, the electric tension-dependent logarithm of the rate constant was fitted with the Arrhenius equation for different membrane potentials. The presence of membrane potential did not lead to any significant changes in the pore edge tension. The increase in electroporation is reasonably explained by the decrease in the prepore free energy barrier. The choice of buffer used in GUVs can significantly influence the kinetics of electroporation. This study provides valuable insights that can contribute to the application of electroporation techniques in the biomedical field.

## 1. Introduction

The electrochemical potential difference between the intracellular and extracellular environments gives rise to a membrane potential (*φ*_m_) that spans a range of −20 to −150 mV [1]. This negative membrane potential plays a crucial role in various cellular processes [2], which include regulating cell proliferation and differentiation [3,4]. It exerts a regulatory influence on the dynamics of phospholipids in the plasma membrane as well as on K-Ras signaling [5]. It also plays a pivotal role in shaping the regulation of dopamine transporter trafficking at the plasma membrane [6].

On the other hand, irreversible electroporation (IRE) is an advanced technique utilized for tissue and cancer cell ablation [7,8]. In this technique, a high-intensity electric field is applied to cells to induce the opening of membrane pores, resulting in a sudden and significant increase in membrane permeability. This technique has gained recognition and is being explored for various medical applications, including the treatment of different types of cancer [9]. It has been well-known that the structure of the cell membrane is highly intricate, and accurately studying the changes in its various components and quantities poses significant challenges [10]. As a result, researchers often rely on mimics of cell membranes, such as lipid membranes formed in vesicles. Giant unilamellar vesicles (GUVs) with sizes comparable to cells have been extensively utilized in numerous experiments [11]. These vesicles provide a convenient model system that allows for real-time observations using optical microscopes. By employing GUVs, researchers gain valuable insights into the behavior and properties of cell membranes under controlled conditions.

When an external electric field is applied, it generates lateral electric tension (*σ*_e_) in the lipid membranes of vesicles. If this tension surpasses a critical threshold, membrane poration occurs, ultimately leading to ablation or cell death. Similarly, mechanical tension (*σ*_m_) can be induced in membranes by applying suction pressure using a micropipette [12]. This technique allows for the controlled manipulation and study of membrane mechanics. Recently, several experiments have been conducted using *σ*_e_ and *σ*_m_ to investigate the effects of varying lipid composition, surface charge density, cholesterol concentration, and osmotic pressure in the absence of *φ*_m_ [13]. These studies aim to understand the impact of these parameters on the kinetics of GUVs and their rupture probability. In living cells, resting membrane potential is maintained across the cell membrane, which is essential for various cellular processes. By studying the effects of different membrane potentials on electric field-induced rupture, researchers aim to gain insights into the mechanisms underlying the response of cells and vesicles to electric fields.

Membrane proteins play crucial roles in essential processes for the survival and function of biological cells. They are involved in mediating the transport of ions and larger solutes across membranes, facilitating communication between the cell and its surroundings through receptors, and catalyzing chemical reactions as membrane-embedded enzymes [14]. In order to generate *φ*_m_ across the membranes of GUVs, a commonly employed method involves the use of a channel-forming protein called Gramicidin A (GrA) [15,16]. It is an antibiotic peptide produced by *Bacillus brevis* that forms inorganic monovalent cation-permeable channels in biological membranes and lipid bilayers. Its small size and ease of chemical modification make it an excellent candidate for investigating the properties of real ion channels, as they share similar structural features [17]. Generally, ion channels regulate ionic permeability [18], and manage the passage of ions in all excitable cell membranes [19].

Membrane potential is vital for the rapid permeabilization of the plasma membrane and lipid bilayers by the antimicrobial peptide lactoferricin B [20]. The application of membrane potentials has been found to have a significant impact on various aspects of membrane behavior, including the effects of antimicrobial peptide magainin 2-induced membrane poration as well as the binding interactions between membranes and magainin 2 [21]. The membrane potential also plays a crucial role in the entry process of lactoferricin B-derived 6-residue antimicrobial peptide and cell-penetrating peptide transportan 10 into single *Escherichia coli* cells and lipid vesicles [22].

Membrane tension is a fundamental physical parameter that presents both challenges and widespread implications in cell biology. Maintaining proper membrane tension is essential for the integrity and functionality of cells [23,24]. Osmotically induced membrane tension plays a crucial role in facilitating the triggering of electropermeabilization in living cells [25,26]. When an external force, such as electrical, mechanical, or optical tension, causes the formation of a pore in the lipid bilayer, it leads to the generation of pore edge tension (Γ) in the membrane [27–30]. The pore edge tension, also known as line tension, is a critical factor that determines the stability of pores formed in the membrane and drives their closure. This tension is intimately linked to membrane stability and has a significant impact on the mechanisms involved in membrane resealing after pore formation. It is an intrinsic characteristic of the membrane that arises from the physicochemical properties and amphiphilic nature of the lipid molecules within the bilayer. This tension reflects the energy cost per unit length required to maintain an open pore in the membrane. The increase of monolayer spontaneous curvature on tension-induced pore formation decreases the Γ, resulting in to an increase in the rupture kinetics of GUVs [31].

Cellular homeostasis relies on the maintenance of a resting membrane potential across cell membranes. Consequently, it is crucial to examine the static and dynamic behavior of cell-mimetic vesicles when exposed to an electric field in the presence of various membrane potentials. Previous studies conducted by the Yamazaki group have explored the impact of membrane potential on vesicles and cells, providing insights into the mechanisms underlying damage induced by antimicrobial peptides and cell-penetrating peptides on lipid bilayers of GUVs and cell membranes [22,32,33]. However, there has been a notable lack of investigations focused on studying the effects of membrane potential on electroporation of lipid vesicles. Therefore, the primary objective of this research is to investigate electric field-induced vesicle electroporation and measure pore edge tension under the influence of various membrane potentials. By examining multiple electroporation regimes in the presence of membrane potential, we aim to comprehensively evaluate the effects of the electric field on biological cells. The outcomes of this research will contribute to a deeper understanding of the underlying mechanisms of electroporation and provide essential information for the effective development of electroporation technique-based biomedical applications.

## 2. Materials and methods

### 2.1 Chemicals and reagents

1,2-dioleoyl-*sn*-glycero-3-phospho-(1´-*rac*-glycerol) (sodium salt) (DOPG), and 1,2-dioleoyl-*sn*-glycero-3-phosphocholine (DOPC) were purchased from Avanti Polar Lipids Inc. (Alabaster, AL). Gramicidin A (GrA) from Bacillus brevis, 4-(2-Hydroxyethyl) piperazine-1-ethanesulfonic acid (HEPES), 1,4-Piperazinediethanesulfonic acid (PIPES), Tetraethylammonium chloride (TEAC), Bovine serum albumin (BSA), and O,O´-Bis (2-aminoethyl) ethyleneglycol-*N*,*N*,*N´*,*N´*, -tetraacetic acid (EGTA), potassium chloride (KCl), sodium chloride (NaCl), glucose, and sucrose were purchased from Sigma-Aldrich (Germany).

### 2.2 Synthesis of GUVs

DOPG/DOPC/GrA (40/60/0.01)-GUVs, where the numbers indicate the molar ratio, were prepared using the natural swelling method [34]. To prepare the samples, we started by adding 80 μL of 1 mM DOPG and 120 μL of 1 mM DOPC, both dissolved in chloroform, into a 5 mL glass vial. After that, it was added 20 μL of 1 μM GrA, which was dissolved in ethanol, to the same vial. The DOPG, DOPC, and GrA components mixed together naturally due to their high diffusion rates in the solvent. Additionally, we gently shook the vial by hand to ensure thorough mixing. At this stage, it was allowed the mixture to sit for approximately 10 minutes. This process was repeated for another vial to prepare a total of two vials for each experiment. Then the solvents were evaporated using a gentle flow of nitrogen gas. To ensure complete drying, the glass vials were placed in a vacuum desiccator connected to a rotary vacuum pump for at least 12 hours. Next, 20 μL of MilliQ water was added to each vial, and the vials were pre-hydrated by incubating them at ~48°C for 8 minutes in a mini water bath. Afterward, 1 mL of buffer A (10 mM HEPES, 150 mM KCl, pH 7.5, and 1 mM EGTA) containing 0.10 M sucrose was added to each vial. The samples were then incubated at 37°C for 2.5 hours. To separate the GUVs from aggregates, the samples were subjected to centrifugation using 13000×*g* (here *g* is the acceleration due to gravity) at 20°C for 20 minutes using a centrifuge machine (NF 800R Centrifuge, Nuve, Turkey). Subsequently, the GUV suspension underwent purification using the membrane filtering method [35]. The purified GUV suspension was transferred to a solution of buffer A containing 0.10 M glucose. The dynamics of the GUVs were observed using a phase contrast microscope (IX 73 Olympus, Japan) with a 20× objective at a temperature of 25 ± 1°C (Tokai Hit Thermo Plate, Japan). The images of the GUVs were recorded using a charged coupled camera (CCD) (DP22, Olympus) at a speed of 25 frames per second.

### 2.3 Generating negative membrane potential (*φ*_m_)

**Fig 1:**
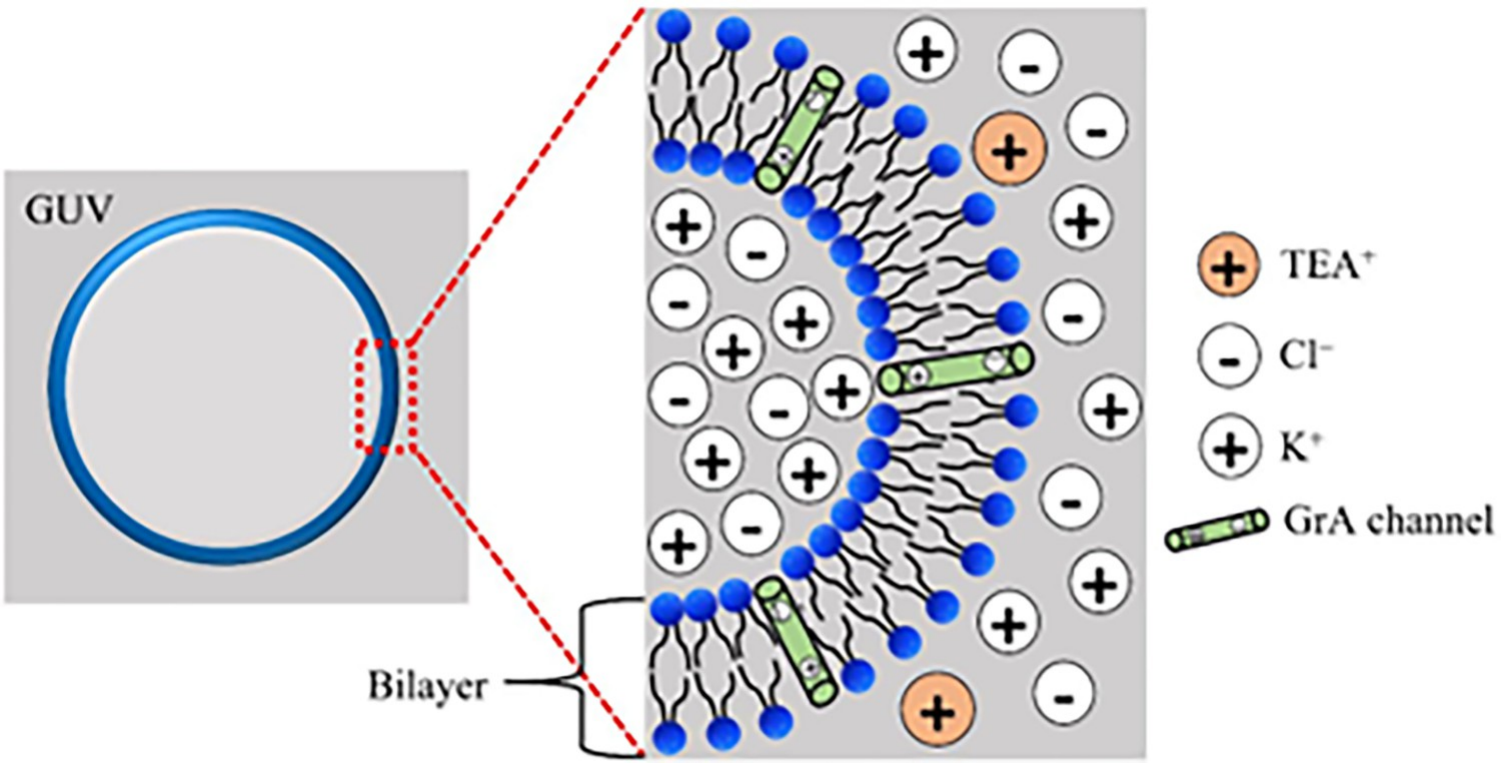
Generating membrane potential across the lipid bilayer. The left side of the figure shows the GUV and the right side shows the concentration of ions across the bilayer.

The purified GUVs were transferred to a microchamber of the volume of 200 μL, and the GUVs were diluted using buffer T (10 mM HEPES, 150 mM TEAC, pH 7.5, and 1 mM EGTA) containing 0.10 M glucose. To prevent any effects of osmotic pressure, a similar osmolarity was maintained by keeping the combined concentration of KCl and TEAC inside and outside of the GUVs. Different concentrations of K^+^ ions were used to establish a concentration gradient between the inside and outside of the membrane, thereby generating various membrane potentials [20]. In order to prevent strong attachment between the GUVs and the glass surface of the microchamber, a pre-coating step was performed using a 0.10% (w/v) solution of BSA. The membrane potential across the membrane was calculated using the Nernst equation [36]:

$$\varphi_{m}=25.7ln\frac{{\left[K^{+}\right]}_{out}}{{\left[K^{+}\right]}_{in}}$$ (1)

where, [K^*+*^]_out_ and [K^*+*^]_in_ are the concentrations of K^*+*^ ions outside and inside the GUVs, respectively. The generation of membrane potential is illustrated in Fig 1.

An example to generate *φ*_m_ = −30 mV is given here. The inside [K^+^]_in_ = 150 mM, and hence the final concentration in the outside of GUV is required, [K^+^]_out_ = 150× e ^-25.7/30^ = 46.67 mM. If the final volume of the microchamber was 200 μL, the required purified GUVs is (200 μL×46.67 mM)/150 mM = 62.23 μL, which was diluted with (200 − 62.23) μL = 137.77 μL of buffer T. The same procedure was followed for generating −60 mV and −90 mV. A study successfully demonstrated the establishment of the resulting membrane potential (*φ*_m_) using a specific technique. In the technique, the intensity of the rim of GUV, as indicated by a membrane potential-sensitive dye 3,3’-dihexyloxacarbocyanine iodide DiOC6(3), was employed as a marker of the membrane potential [20]. The study examined the interaction between 2 nM DiOC6(3) and individual DOPG/DOPC/GrA (40/60/0.01)-GUVs in the presence of various membrane potential conditions. We utilized the relevant literature and employed the Nernst equation to calculate the membrane potential in our experiment.

### 2.4 Electric field-induced membrane tension

After 10 min of transferring GUVs and buffer T into the microchamber, an electric field (*E*) was applied to the GUVs. When an electric field is applied to GUVs, transmembrane voltage (*V*_m_) is generated across the lipid bilayer. Such voltage leads to induce the lateral electric tension *σ*_c_ in the membrane as follows [37]:

$$\sigma_{e}=\varepsilon_{m}\varepsilon_{0}\left(\frac{h}{2h_{e}^{2}}\right)V_{m}^{2}$$ (2)

In the equation, *ε*_m_ represents the membrane permittivity (~ 4.5), *ε*_0_ is the permittivity of free space, *h* is the thickness of the membrane (~ 4 nm), and *h*_e_ is the membrane dielectric thickness (~ 2.8 nm). When a spherical GUV is exposed to an *E*, the angle (*θ*) between the direction of *E* and the normal to the bilayer surface can range from 0 to 90°. This means that the orientation of *E* relative to the GUV’s membrane can vary throughout the vesicle’s surface. The relationship between *V*_m_ and *θ* can be expressed as: *V*_m_ = 1.5*RE*|cosθ|. The value of *V*_m_ is maximum when *θ* = 0°, corresponding to *V*_m_ = 1.5*RE*, which is referred to as the "critical membrane voltage for the breakdown of vesicle" (*V*_c_). By substituting the value of *V*_m_ in Eq (2), the following equation can be obtained [38]:

$$\sigma_{e}=22.86\;R^{2}E^{2}\;(mN/m)$$ (3)

where, *R* is the radius of the GUVs [m], *E* is applied electric field [V/m]. Thus, the tension at which a GUV breaks down is influenced by both the applied *E* and the size of the GUV. If *R* = 10 μm and *E* = 553 V/cm, *V*_m_ = 0.83 V and *σ*_e_ = 7 mN/m.

### 2.5 Experimental setup to apply the electric field on the GUVs

**Fig 2:**
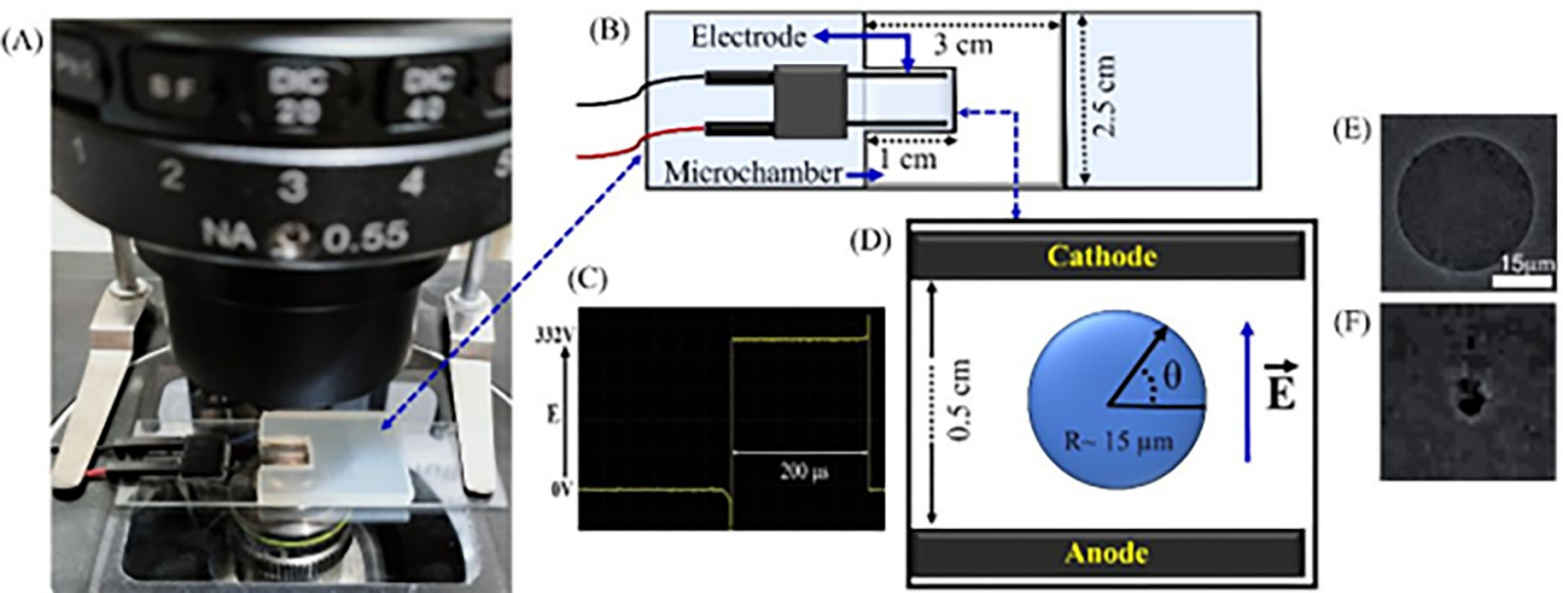
Experimental setup to apply the electric field to the GUVs. (A) Laboratory set up of a microchamber at the stage of a microscope. (B) Schematic representation of the microchamber and gold-coated electrode with proper configuration. The microchamber was fabricated by placing a U-shaped silicone rubber spacer onto a glass slide. (C) Pulsating DC of frequency 1.1 kHz. (D) A ‘single GUV’ between the gold-coated electrodes, where *E* indicates the electric field. Phase contrast image of (E) intact GUV and (F) ruptured GUV.

We examined the effects of electric field on a ‘single GUV’ placed in solutions with different membrane potentials (e.g., *φ*_m_ = 0, −30, −60 and −90 mV). The experimental setup for applying the electric field is presented in Fig 2(A). The size of the microchamber and electrode configuration is shown in Fig 2(B). A pulsating DC signal of frequency 1.1 kHz is used in the investigations (Fig 2C). Usually, vesicle suspension contains different sizes of GUVs. We examined a ‘single GUV’ in each microchamber, in which the size range of GUVs was 30−35 μm. To obtain statistically reliable results, we repeated the same experiment several times by examining different ‘single GUVs’ in different microchambers. To apply an electric field to the GUVs, first measured the size of each selected ‘single GUV’, and then determined the required electric tension using Eq (3). The corresponding range of *E* was 250–450 V/cm. The electric tension *σ*_e_ was applied for a maximum time 60 s. Fig 2(D) shows an illustration of a ‘single GUV’ between the two gold-coated electrodes. The time when the GUV was completely ruptured is defined as the time of pore formation. Before applying the electric field to the GUVs shown in Fig 2(E) and after rupture of the same GUV shown in Fig 2(F).

## 3. Experimental results

We conducted a comprehensive investigation on the effects of negative membrane potential (*φ*_m_) on the electroporation of lipid vesicles. We varied the membrane potential, electric tension (*σ*_e_), and lipid composition to study their effects. Initially, we explored the electroporation of DOPG/DOPC/GrA (40/60/0.01)-GUVs under different *φ*_m_ while maintaining a fixed *σ*_e_. Subsequently, we examined the electroporation of the DOPG/DOPC/GrA (40/60/0.01)-GUVs under various *σ*_e_ while keeping the *φ*_m_ constant. We calculated the rate constant of rupture (*k*_r_), probability of rupture (*P*_rup_), and the average time of intact GUVs (*t*_intact_) under these specified conditions. Furthermore, we examined the electroporation of DOPG/DOPC (40/60)-GUVs in HEPES and PIPES buffer. The obtained results were compared with each other for further analysis and insights.

### 3.1 Electroporation in DOPG/DOPC/GrA (40/60/0.01)-GUVs in the presence of various membrane potentials

**Fig 3:**
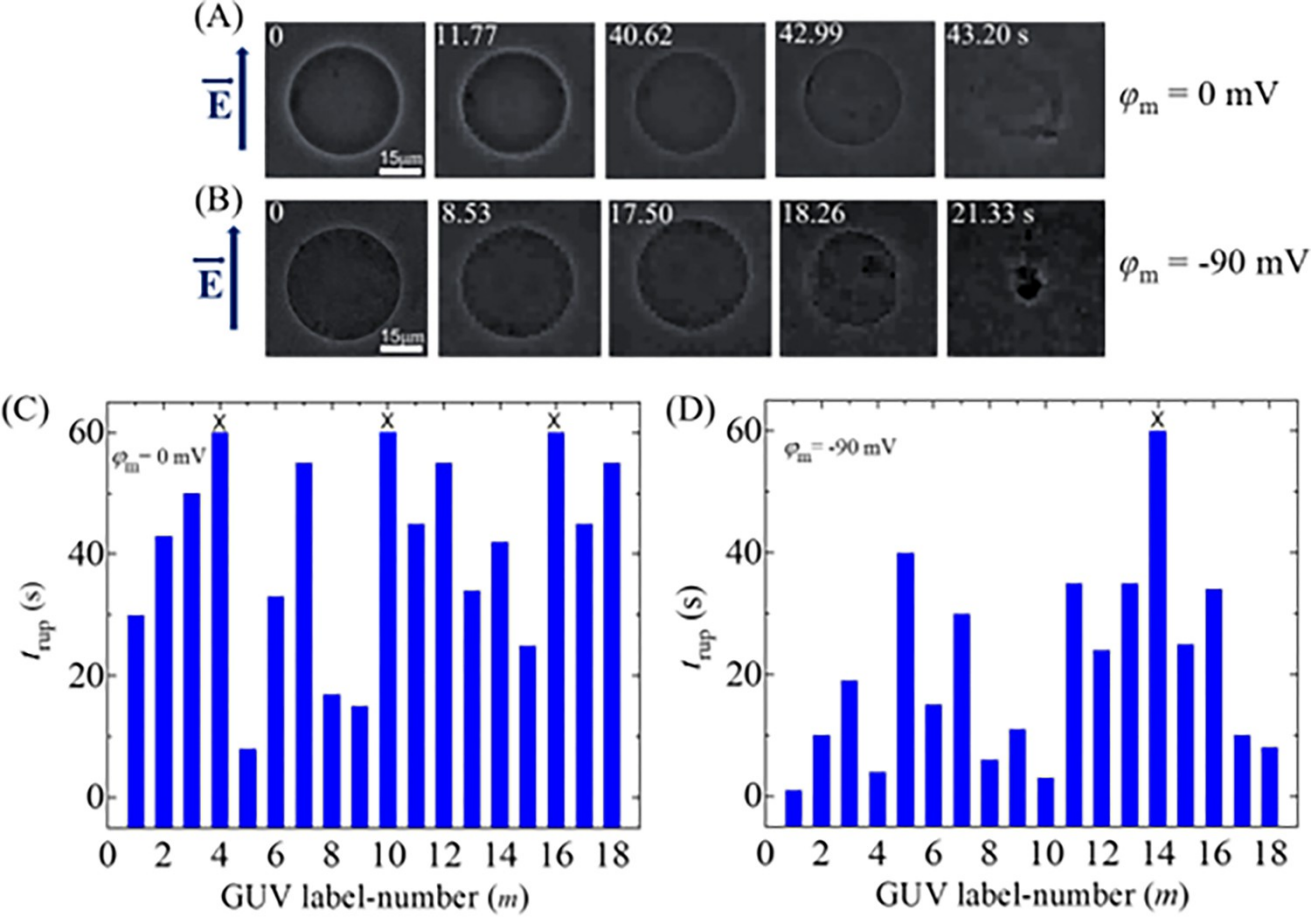
Rupture of DOPG/DOPC/GrA (40/60/0.01)-GUVs for 0 and −90 mV at *σ*_e_ = 6 mN/m. Phase contrast images of rupture of a ‘single DOPG/DOPC/GrA (40/60/0.01)-GUV’ at (A) *φ*_m_ = 0, and (B) *φ*_m_ = −90 mV. The electric field (*E*) direction is shown with an arrow on the left side. The numbers in each image show the time in seconds after applying *E*. The stochastic rupture of several ‘single GUVs’ at (C) *φ*_m_ = 0, and (D) *φ*_m_ = −90 mV.

At first, we investigated the effects of membrane potential (*φ*_m_) on the electroporation in DOPG/DOPC/GrA (40/60/0.01)-GUVs. Fig 3 shows the experimental results of the rupture of GUVs at 0 and −90 mV at *σ*_e_ = 6 mN/m. Without applying electric field (i.e., 0 s), the GUVs become intact and spherical in shape, as shown in Fig 3(A) and 3(B). At *φ*_m_ = 0, the GUV remains intact until 40.62 s (Fig 3A). GUV starts to rupture at 42.99 s, and at 43.2 s, it becomes completely ruptured as the structure of the GUV disappears. Similarly, at *φ*_m_ = −90 mV, GUV is started to rupture at 18.26 s (Fig 3B). The rupture occurs due to the rapid increase in the radius of the pores formed in the GUV membranes. The starting time of rupture indicates the time of pore formation, signifying the point at which the structural integrity of the GUV disappears.

The same experiments were carried out for several ‘single GUVs’ (number of examined GUVs was *N* = 12−18) in each independent experiment (number of independent experiments was *n* = 2−4) under the same conditions. The rupture time (*t*_rup_) for several GUVs in one independent experiment is different both for 0 mV and −90 mV, indicating the stochastic nature of rupture (Fig 3C and 3D). The required rupture time of several GUVs at 0 mV is relatively larger compared to −90 mV. In Fig 3(C), 15 GUVs rupture out of 18 GUVs at 0 mV, whereas 17 GUVs rupture at −90 mV (Fig 3D). The time required to rupture the GUVs is defined as the rupture time of vesicles (*t*_rup_). A ‘single GUV’ was observed until 60 s, whether the GUV ruptured or not. If any GUVs didn’t rupture within 60 s, they are considered intact and indicated by a symbol (×) mark at the top of the bar (Fig 3C and 3D). A similar rupture along with stochastic rupture of DOPG/DOPC/GrA (40/60/0.01)-GUVs is also observed for −30 mV and −60 mV at *σ*_e_ = 6 mN/m.

**Fig 4:**
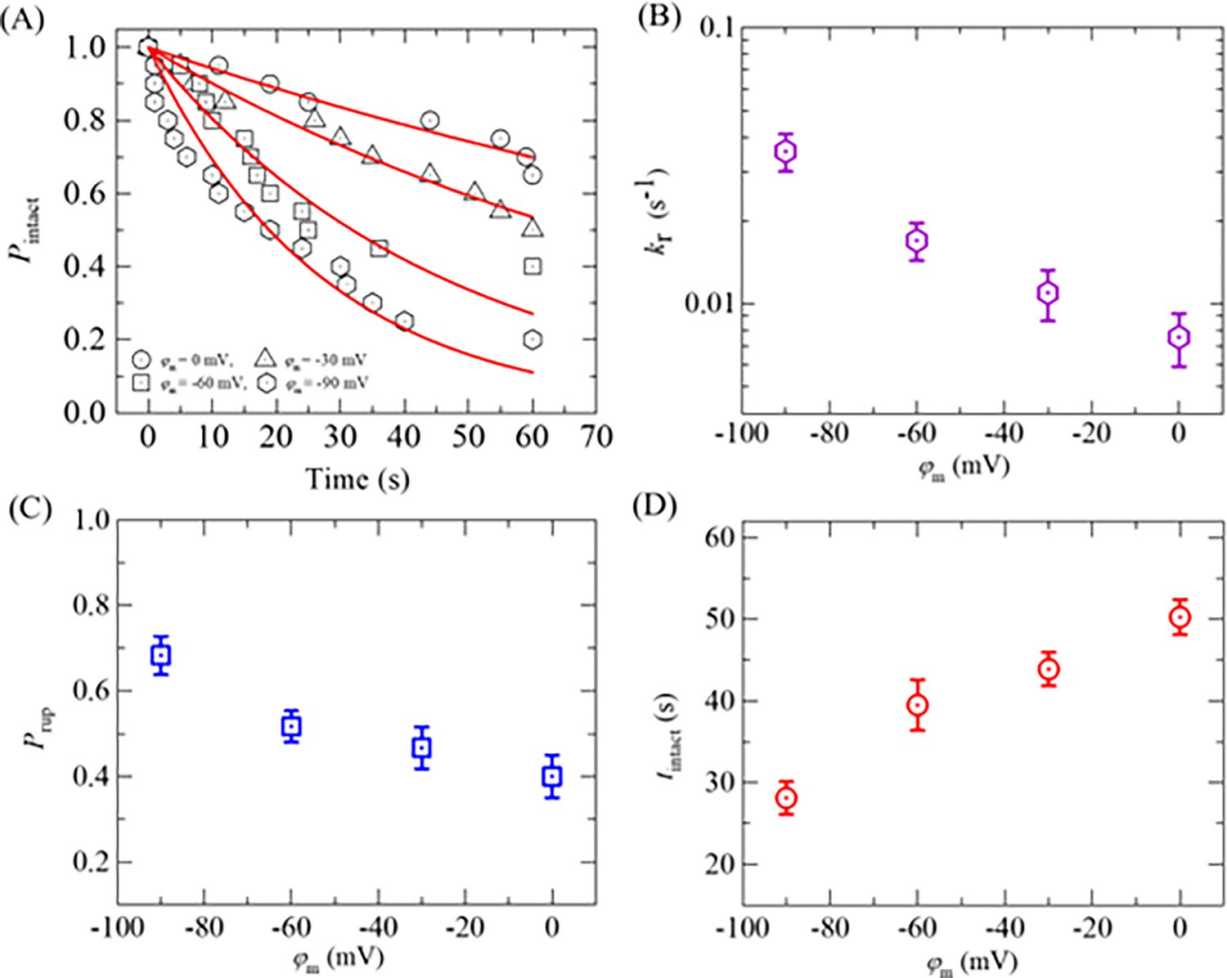
Rate constant of rupture, probability of rupture, and the average time of intact DOPG/DOPC/GrA (40/60/0.01)-GUVs in the presence of various *φ*_m_ at 6 mN/m. (A) Time course of *P*_intact_ for 0, −30, −60, and −90 mV. The membrane potential dependent (B) rate constant, (C) probability, and (D) average time of intact GUVs.

To determine the rate constant of rupture of GUV (*k*_r_) for different negative membrane potentials at a fixed *σ*_e_, the time course of the fraction of intact GUVs without rupture among all the examined GUVs, *P*_intact_ (*t*), is determined, which indicates on how much fraction of GUVs became intact with time and is represented as *P*_intact_ (t) = 1- *P*_rup_ (*t*). If 18 single GUVs are examined at *σ*_e_ = 6 mN/m in which 9 GUVs rupture within 60 s observation, *P*_intact_ (*t*) = 1- *P*_pore_ (*t*) = 1−9/18 = 0.5. Fig 4(A) shows the time course of *P*_intact_ (*t*) for different membrane potentials at 6 mN/m. It shows that the decrement of experimental data for *P*_intact_ (t) vs. time is a factor when membrane potential changes from 0 to −90 mV. The *P*_intact_ (*t*) vs. time graph is well fitted by a single-exponential decay function,

$$P_{intact}(t)=\exp\left(-k_{r}t\right)$$ (4)

where, *t* is the duration of time to apply the tension in GUVs. From the fitted curves, the values of *k*_r_ are obtained 6.2×10^−3^, 10.4×10^−3^, 21.8×10^−3^, and 36.7×10^−3^ s^-1^ for 0, −30, −60 and −90 mV, respectively. The same experiments were carried out for several ‘single GUVs’ (*N* = 12−18) in each independent experiment (*n* = 2−4). The values of average *k*_r_ are (7.5 ± 1.6)×10^−3^, (10.9 ± 2.3)×10^−3^, (16.9 ± 2.6)×10^−3^, and (35.6 ± 5.5)×10^−3^ s^-1^ for 0, −30, −60 and −90 mV, respectively, at 6 mN/m. Hence, the values of *k*_r_ increase with the increase of negative membrane potential (Fig 4B).

We have calculated the probability of rupture (*P*_rup_) and the average time of intact (*t*_intact_) of DOPG/DOPC/GrA (40/60/0.01)-GUVs for different *φ*_m_ at 6 mN/m. The values of *P*_rup_ were 0.41 ± 0.05, 0.47 ± 0.02, 0.52 ± 0.02, and 0.68 ± 0.04 for 0, −30, −60, and −90 mV, respectively (Fig 4C). *P*_rup_ increases with the increase of *φ*_m_. The average times of intact GUVs (*t*_intact_) are 50.25 ± 2.1, 43.88 ± 2.0, 39.50 ± 3.1, and 28.11 ± 2.0 s for 0, −30, −60, and −90 mV, respectively, at 6 mN/m. Less intact time of GUVs is observed in higher *φ*_m_ (Fig 4D).

Now, we quantify the change in rate constant of rupture, probability of rupture, and the average time of intact DOPG/DOPC/GrA(40/60/0.01)-GUVs at *σ*_e_ = 6 mN/m with the change in membrane potential. Table SI 1 in S1 File represents the values of different parameters in the presence of *φ*_m_. Table SI 2 in S1 File is extracted from Table SI 1 in S1 File, which presents the changes in *k*_r_, *P*_rup_, and *t*_intact_ due to the changes in *φ*_m_. The average values of Δ*k*_r_/Δ*φ*_m_, Δ*P*_rup_/Δ*φ*_m_, and |Δ*t*_intact_|/Δ*φ*_m_ are obtained 1.1×10^−4^ (s^-1^/mV), 2.3 (mV^−1^), and 22.3×10^−2^ (s/mV), respectively, at *σ*_e_ = 6 mN/m and at *φ*_m_ = −30 mV.

### 3.2 Electroporation in DOPG/DOPC/GrA (40/60/0.01)-GUVs in the presence of various electric tensions at *φ*_m_ = −30 mV

**Fig 5:**
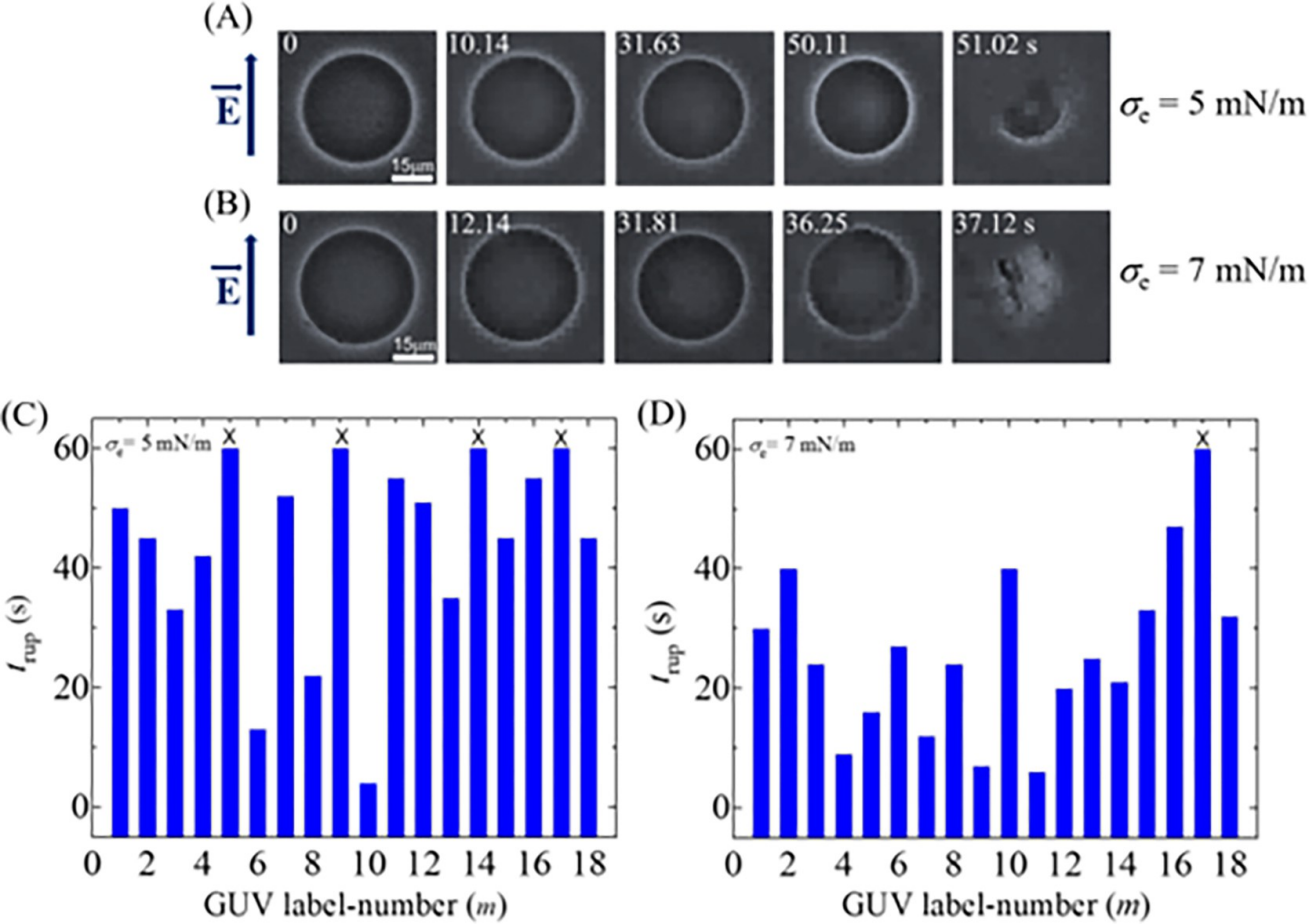
Rupture of DOPG/DOPC/GrA (40/60/0.01)-GUVs at 5 and 7 mN/m in the presence of −30 mV. Phase contrast images of rupture of a ‘single DOPG/DOPC/GrA (40/60/0.01)-GUV’ at (A) *σ*_e_ = 5 mN/m and (B) *σ*_e_ = 7 mN/m. The electric field (*E*) direction is shown with an arrow on the left side. The numbers in each image show the time in seconds after applying *E*. The stochastic rupture of several ‘single GUVs’ at (C) *σ*_e_ = 5 mN/m, and (D) *σ*_e_ = 7 mN/m.

In this section, we have investigated the electric tension (*σ*_e_) dependent electroporation in DOPG/DOPC/GrA (40/60/0.01)-GUVs at *φ*_m_ = −30 mV, and presented the results in Fig 5. The phase contrast images of two separate ‘single GUVs’ at 5 and 7 mN/m are presented in Fig 5(A) and 5(B), respectively in the presence of *φ*_m_ = -30 mV. Both the GUVs remain intact and spherical before applying the electric filed (i.e., 0 s). The complete rupture of these GUVs occurs at 51.02 and 37.12 s at 5 and 7 mN/m, respectively. The stochastic rupture of several ‘single GUVs’ for both conditions is shown in Fig 5C and 5D. The unruptured GUVs are indicated by (×) mark at the top of the respective bar diagram. The number of ruptured GUVs at 5 mN/m is relatively smaller compared to 7 mN/m.

**Fig 6:**
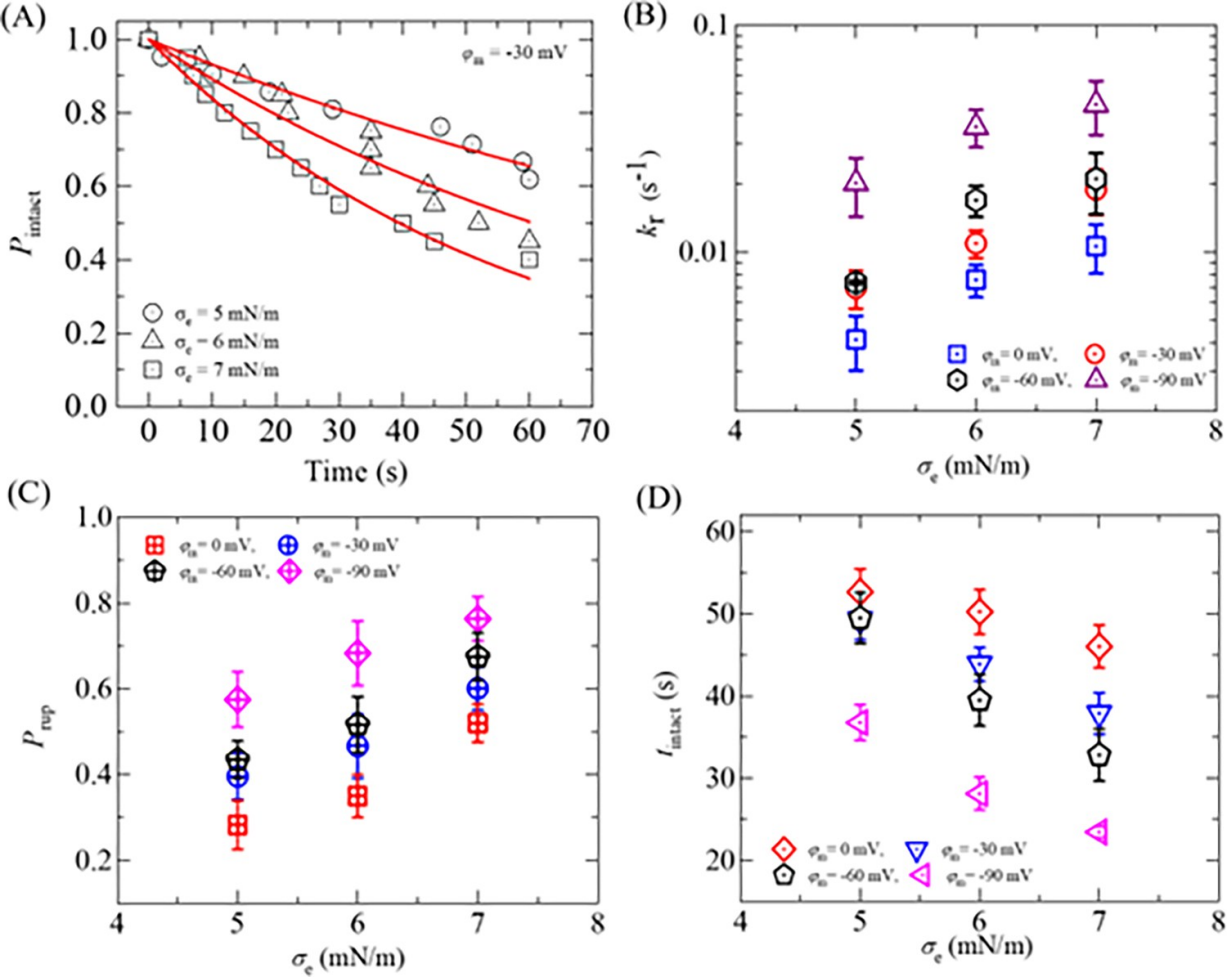
Rate constant of rupture, probability of rupture, and the average time of intact DOPG/DOPC/GrA (40/60/0.01)-GUVs in the presence of various *φ*_m_ and *σ*_e_. (A) Time course of *P*_intact_ for 5, 6, and 7 mN/m at *φ*_m_ = −30 mV. The *σ*_e_ dependent (B) rate constant, (C) probability, and (D) average time of intact GUVs for different *φ*_m_.

Now, we determine the values of *k*_r_ of DOPG/DOPC/GrA (40/60/0.01)-GUVs under various *σ*_e_. We follow the same procedure as in Section 3.1 for determining the *k*_r_. The experimental data on the time course of *P*_intact_ for various *σ*_e_ are presented in Fig 6(A). The experimental data for each tension is fitted using Eq (4), and the values of *k*_r_ are obtained as 7.1×10^−3^, 10.4×10^−3^, and 17.9×10^−3^ s^-1^ for 5, 6 and 7 mN/m, respectively. The average values of *k*_r_ (*N* = 18−20, *n* = 2−4) are obtained as (7.1 ± 1.3)×10^−3^, (10.4 ± 1.5)×10^−3^, and (17.9 ± 4.3)×10^−3^ s^-1^ for 5, 6, and 7 mN/m, respectively, at −30 mV. It shows the increasing of *k*_r_ with the increase of *σ*_e_ at −30 mV (Fig 6B). Now, we compare these rate constants with those obtained at 0, −60, and −90 mV. At 5 mN/m, the *k*_r_ values are (4.1 ± 1.2)×10^−3^, (7.1 ± 1.3)×10^−3^, and (20.1 ± 5.8)×10^−3^ s^-1^ for 0, −30, and −90 mV, respectively. The similar differences in *k*_r_ for different *φ*_m_ are also observed at 6 and 7 mN/m (Fig 6B). Thus, at a fixed tension *σ*_e_, *φ*_m_ greatly increases the kinetics of vesicle rupture. We have calculated the probability of rupture (*P*_rup_) for various *φ*_m_ and *σ*_e_. At *σ*_e_ = 6 mN/m, the values of *P*_rup_ are found as 0.35 ± 0.05, 0.47 ± 0.07, and 0.68 ± 0.07 for 0, −30, and −90 mV, respectively. The same trend of increasing the values of *P*_rup_ with *φ*_m_ is obtained for 5 and 7 mN/m. The *σ*_e_ dependent *P*_rup_ for various *φ*_m_ is presented in Fig 6(C), indicating that the presence of higher *φ*_m_ increases the probability of rupture of GUVs irrespective of membrane tension. In a similar way, the values of the average time of intact (*t*_intact_) are obtained as 37.4 ± 4.8, 29.7 ± 2.8, and 19.8 ± 2.7 s for 5, 6, and 7 mN/m, respectively, at −30 mV. At 0 mV, the values of *t*_intact_ are obtained 41.4 ± 2.8, 35.4 ± 2.7, and 29.2 ± 2.4 s for 5, 6, and 7 mN/m, respectively. Similarly, at −90 mV, these values are obtained as 19.6 ± 2.7, 14.5 ± 3.0, and 13.7 ± 2.2 s for the corresponding tensions. It is observed that less time is required for vesicle rupture at higher *φ*_m_ at a particular tension (Fig 6D).

### 3.3 Estimation of pore edge tension (Γ) of DOPG/DOPC (40/60)-GUVs under various conditions

**Fig 7:**
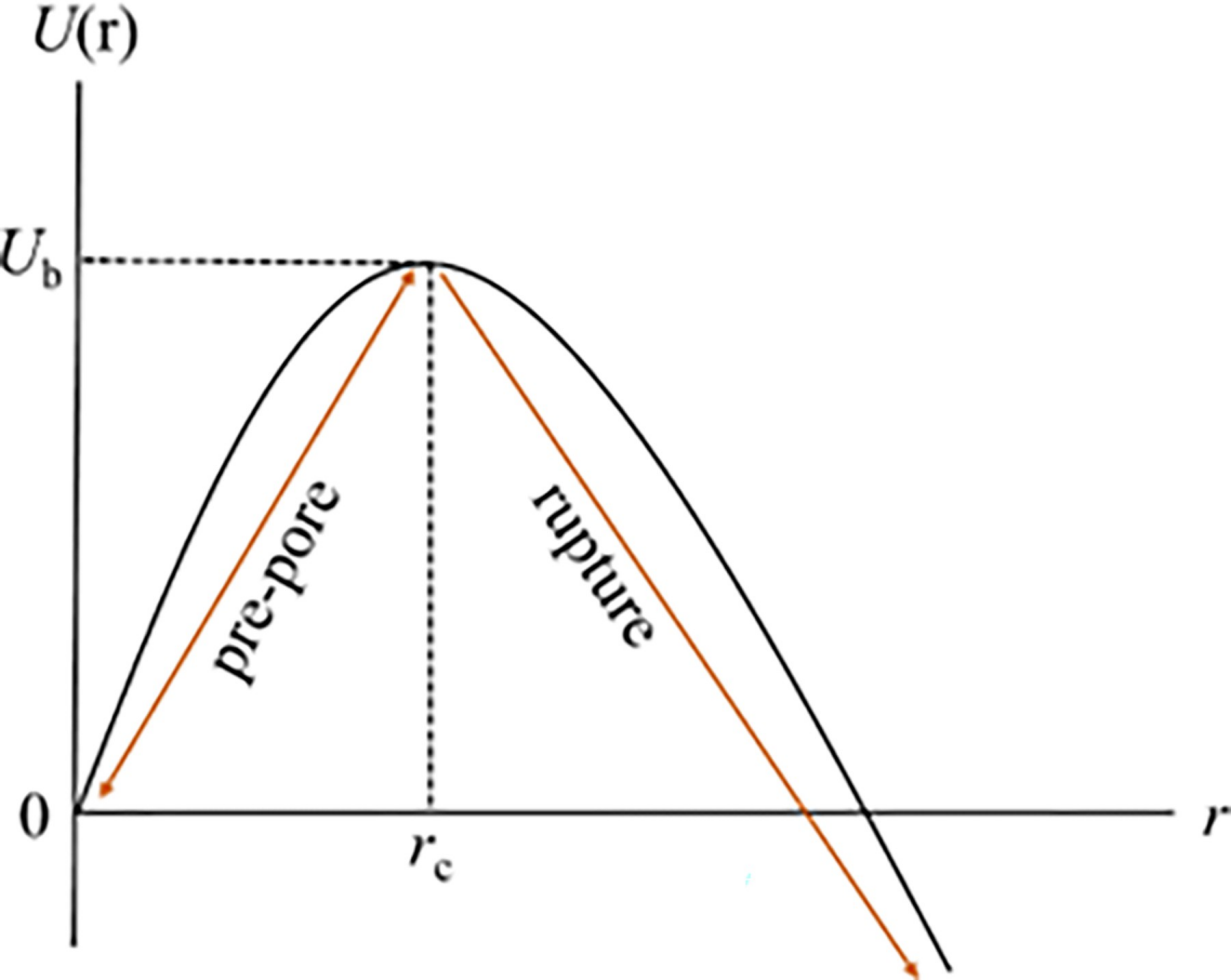
Prepore energy profile. The energy landscape consists of prepore region and the rupture region.


Lipid membranes, composed of lipid molecules, exhibit continuous fluctuations in their lateral density. These fluctuations can lead to localized regions of decreased lipid density known as prepores or local density rarefactions [39,40]. The presence of an electric field (*E*) induces lateral tension (*σ*_e_) in the membranes. Thermal energy further contributes to variations in the lateral density of lipid molecules, causing local condensation and rarefaction within the membrane [38]. When a rarefaction in the membrane exceeds a critical radius, *r*_c_, it transitions into a prepore with a radius, *r*. If *r* is smaller than *r*_c_, the prepore closes rapidly. Conversely, if *r* is greater than or equal to *r*_c_, the prepore transforms into a transmembrane pore. These phenomena are depicted and visualized in Fig 7. Vesicle rupture occurs within a very short time, approximately 1 s, as the radius, *r*, approaches infinity. The free energy of a prepore, *U*(*r*, *σ*_e_), comprises a term -π*r*^2^*σ*_e_ that favor prepore expansion and the term 2π*r*Γ that favor prepore closure, where Γ represents the free energy per unit length of a prepore (i.e., pore edge tension or line tension of a pore). According to the classical theory of pore formation, the free energy of a prepore can be expressed as [41]:

$$U(r,\;\sigma_{e})=2\pi r\Gamma -\pi (\sigma_{e}+B)r^{2}$$ (5)

In our previous paper [42], a similar equation for the free energy of a prepore in the context of IRE was utilized. In that study, we specifically considered the toroidal structure of the prepore [43]. The parameter *B* represents the electrostatic interaction arising from the surface charge of membrane. In our case, *B* is considered to be 1.76 mN/m since the surface charge density of the DOPG/DOPC/GrA (40/60/0.01)-GUVs and the ion concentration in the buffer match those of DOPG/DOPC (40/60)-GUVs [41]. The energy barrier, or activation energy, for pore formation is determined by the maximum value of *U*(*r*) at *r* = *r*_c_, denoted as *U*_b_. At *r*_c_ = Γ/ (*σ*_e_ + *B*), *U*_b_, is expressed as follows [44,45]:

$$U_{b}=\frac{{\pi \Gamma }^{2}}{\left(\sigma_{e}+B\right)}$$ (6)

The Arrhenius equation for the rate constant (*k*_r_) can be derived from Eq (6) and can be expressed as follows [46]:

$$k_{r}=A\;\exp\left[-\frac{\frac{\pi \Gamma^{2}}{(\sigma_{e}+B)}}{k_{B}T}\right]$$ (7)

where, *A* is the frequency factor, *k*_B_ is the Boltzmann constant, and *T* is the absolute temperature. By rearranging Eq (7), we can derive the revised expression as follows:

$$\ln\;k_{r}=\ln\;A-\frac{\pi \Gamma^{2}}{k_{B}T}\left(\frac{1}{(\sigma_{e}+B)}\right)=C-\frac{\pi \Gamma^{2}}{k_{B}T}\left(\frac{1}{(\sigma_{e}+B)}\right)$$ (8)

**Fig 8:**
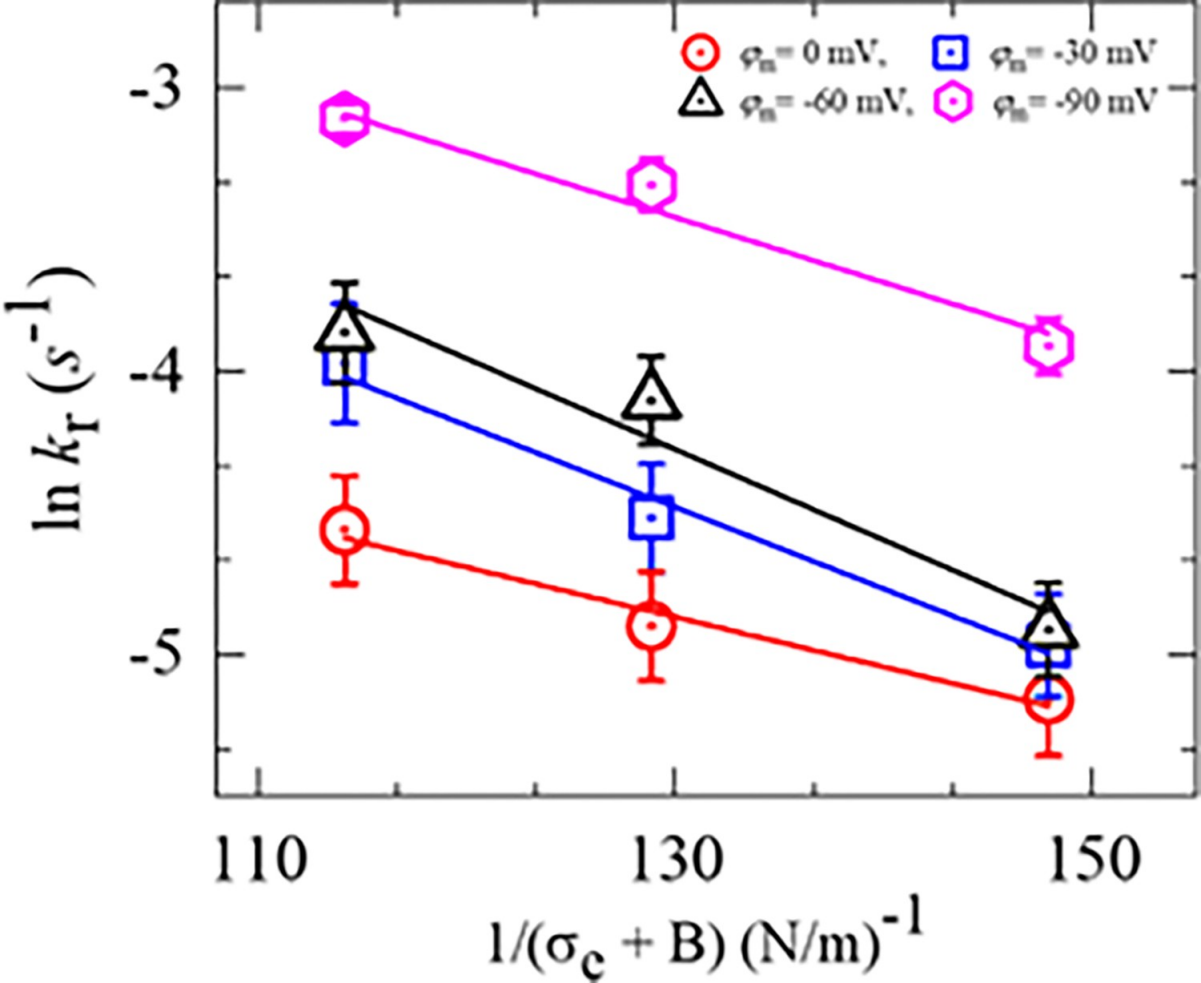
The 1/(*σ*_e_ +*B*) dependent ln*k*_r_ of DOPG/DOPC/GrA (40/60/0.01)-GUVs in the presence of various *φ*_m_. The solid line is the best-fit theoretical curve of Eq (8).

The experimental data on ln*k*_r_ vs. 1/(*σ*_e_ + *B*) for several membrane potentials are fitted using Eq (8) as shown in Fig 8. The best-fit values were obtained for Γ = 4.80 ± 1.98, 6.14 ± 2.23, 6.47 ± 3.18, and 5.48 ± 2.12 pN at 0, −30, −60, and −90 mV respectively. The change in Γ is negligible and often unchanged with respect to the change of *φ*_m_. Therefore, it can be assumed that the value of Γ is almost same for DOPG/DOPC/GrA (40/60/0.01)-GUVs with the change in *φ*_m_.

### 3.4 Electroporation in DOPG/DOPC (40/60)-GUVs in HEPES and PIPES buffer in the absence of membrane potential

**Fig 9:**
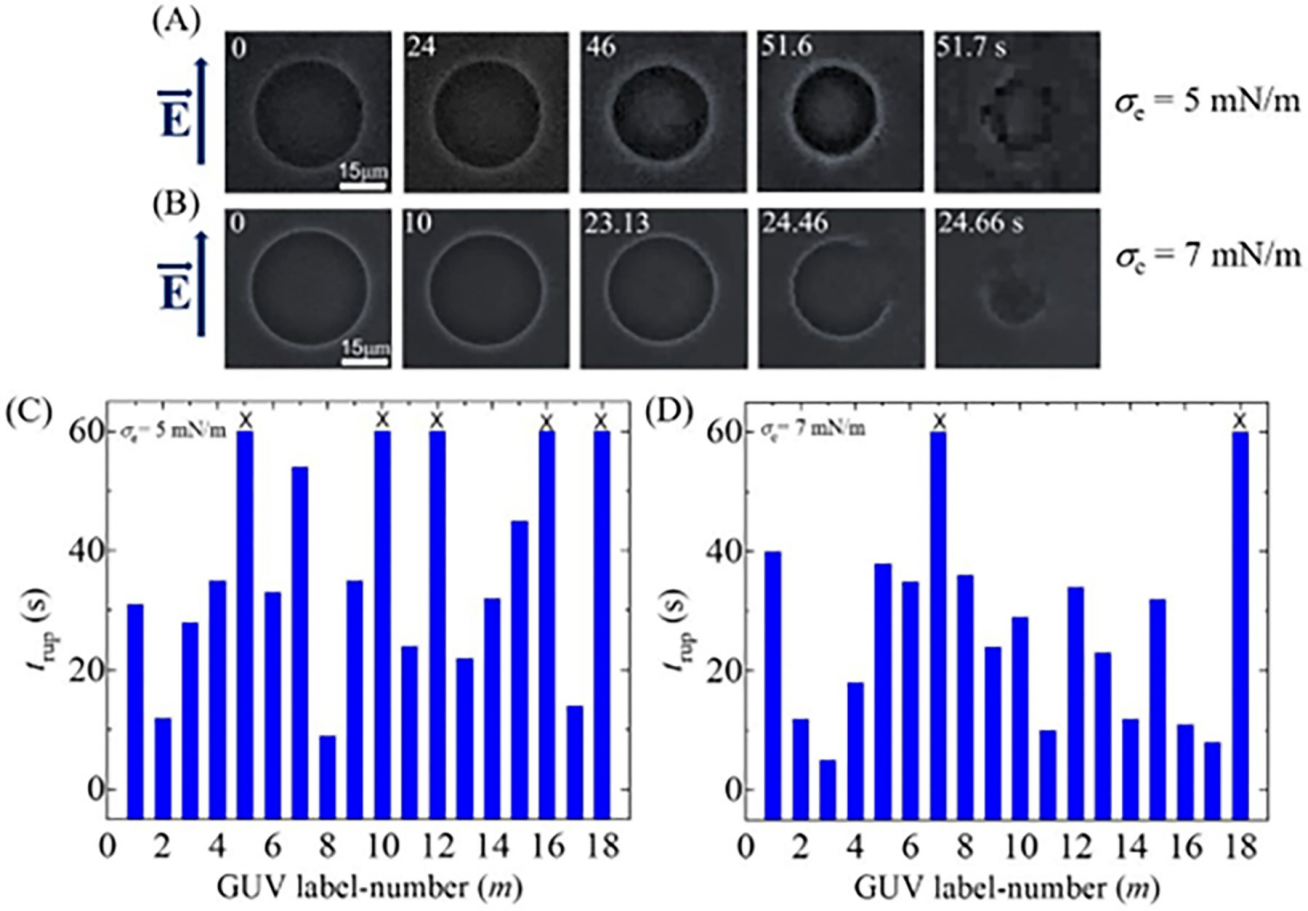
Rupture of DOPG/DOPC (40/60)-GUVs in HEPES buffer. Phase contrast images of rupture of a ‘single DOPG/DOPC (40/60)-GUV’ at (A) *σ*_e_ = 5 mN/m, and (B) *σ*_e_ = 7 mN/m. The electric field (*E*) direction is shown with an arrow in the left side. The numbers in each image show the time in seconds after applying *E*. The stochastic rupture of several ‘single GUVs’ at (C) *σ*_e_ = 5 mN/m, and (D) *σ*_e_ = 7 mN/m.

In this experiment, the electric field-induced rupture of DOPG/DOPC (40/60)-GUVs prepared in HEPES buffer (10 mM HEPES, 150 mM KCl, pH 7.5, and 1 mM EGTA) is investigated in the absence of membrane potential. Fig 9 shows the experimental results of GUV’s rupture under different tensions. Initially, at 0 s, both GUVs (shown in Fig 9(A) and 9(B)) remain intact and exhibit a spherical shape with high contrast in the phase contrast image, indicating their stability. However, when an electric tension of *σ*_e_ = 5 mN/m is applied to the GUV shown in Fig 9(A), rupture occurs at 51.6 s. Similarly, when a higher electric tension of *σ*_e_ = 7 mN/m is applied to the GUV shown in Fig 9(B), rupture occurs at 24.46 s. The stochastic rupture of several GUVs at *σ*_e_ = 5 mN/m and *σ*_e_ = 7 mN/m is presented in Fig 9C and 9D, in which the horizontal axis indicates the GUV label number (*m*) and the vertical axis indicates the rupture time (*t*_rup_). The GUVs that did not rupture within this time frame is indicated by the cross (×) mark on top of the bar (Fig 9C and 9D).

**Fig 10:**
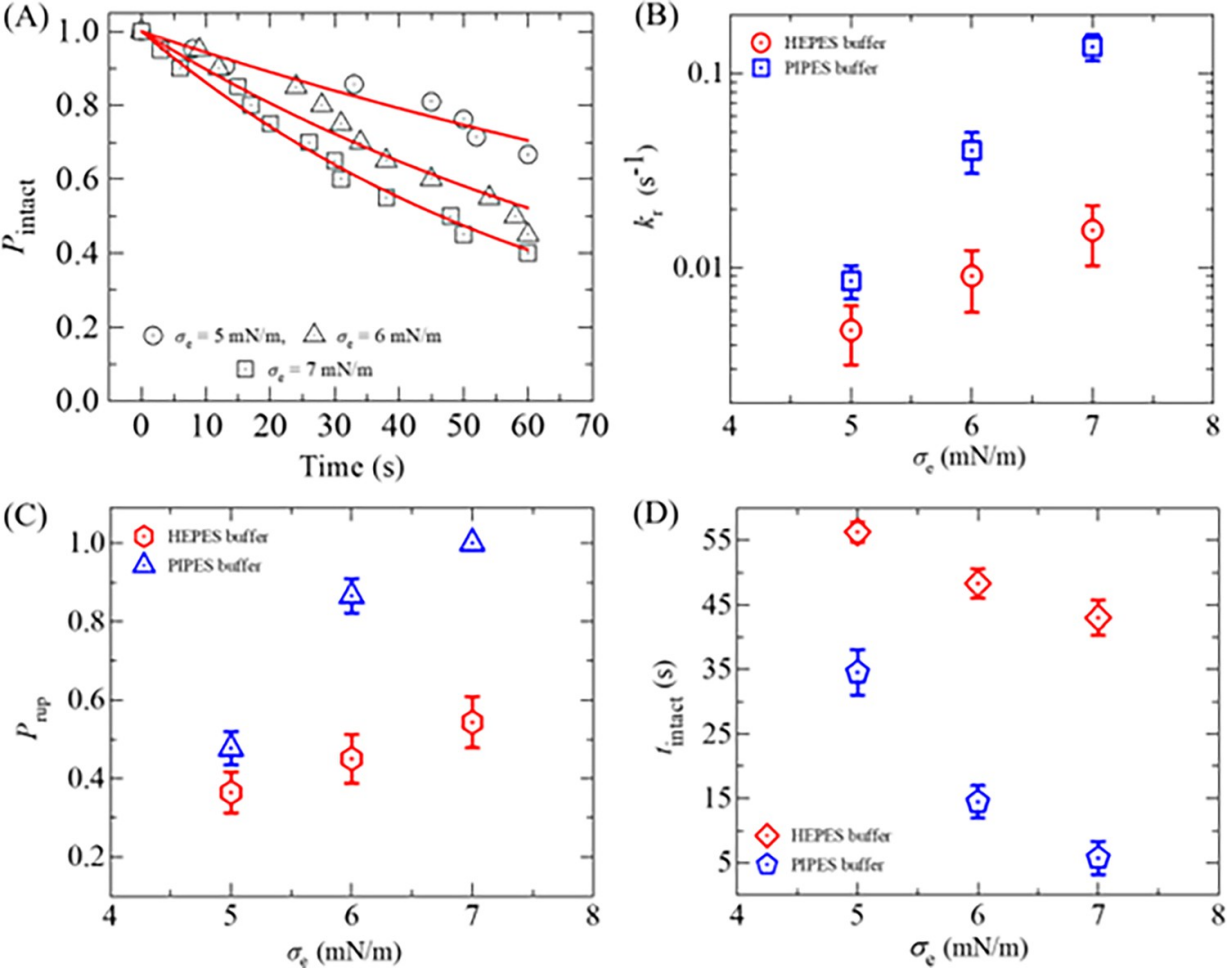
Rate constant of rupture, probability of rupture, and the average time of intact DOPG/DOPC (40/60)-GUVs prepared in HEPES buffer. (A) Time course of *P*_intact_ for various *σ*_e_. The *σ*_e_ dependent (B) rate constant, (C) probability, and (D) average time of intact GUVs.

To determine the *k*_r_ of DOPG/DOPC (40/60)-GUVs, the time-dependent *P*_intact_ is shown in Fig 10(A). The *P*_intact_ vs. time graph is fitted using Eq 4, and the *k*_r_ value is obtained 5.8×10^−3^ s^-1^ for 5 mN/m. Similarly, the values of *k*_r_ are 10.4×10^−3^ s^-1^ and 15.1×10^−3^ s^-1^ for 6 and 7 mN/m, respectively. The average values of *k*_r_ with (± SE) from the several independent experiments (e.g., *N* = 12−18, *n* = 2−4) are obtained (4.8 ± 1.6)×10^−3^ s^-1^, (9.1 ± 3.2)×10^−3^ s^-1^ and (16 ± 5.4)×10^−3^ s^-1^ for 5, 6, and 7 mN/m, respectively (Fig 10B). This is clearly indicating the increasing rate constant with applied field. The values of *P*_rup_ are obtained 0.36 ± 0.05, 0.45 ± 0.06, and 0.54 ± 0.06 for 5, 6, and 7 mN/m, respectively (Fig 10C). It is found that the *P*_rup_ increases with the increase in applied tension. As mentioned above, all the examined GUVs were not ruptured in some cases. The average time of intact (*t*_intact_) GUVs were 56.32 ± 1.6, 48.33 ± 2.3, and 43.03 ± 2.8 s for 5, 6, and 7 mN/m, respectively (Fig 10D). The intact time is comparatively shorter at higher membrane tensions.

**Fig 11:**
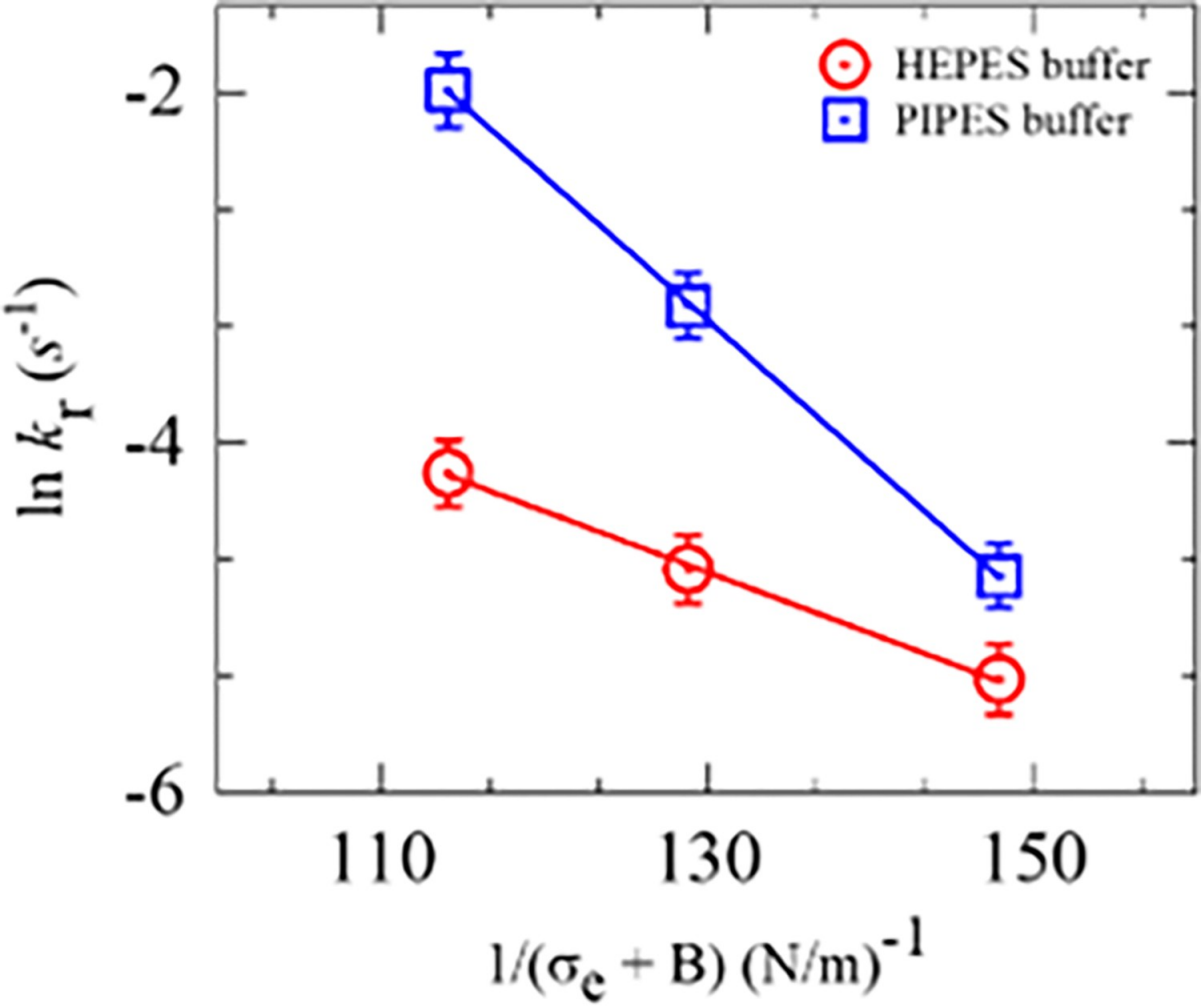
The 1/(*σ*_e_ +*B*) dependent ln*k*_r_ of DOPG/DOPC (40/60)-GUVs obtained for HEPES and PIPES buffer. The solid line is the best-fit theoretical curve of Eq (8).

We also performed the similar experiments in PIPES buffer (10 mM PIPES, 150 mM NaCl, pH 7.5, and 1 mM EGTA) and the results follow the same trend as those obtained in HEPES buffer (Fig 11). The values of *k*_r_, *P*_rup_ and *t*_intact_ at *σ*_e_ = 5 mN/m in PIPES buffer are obtained (8.5 ± 1.7)×10^−3^ s^-1^, 0.36 ± 0.05, and 34.5 ± 3.5 s, respectively, whereas the corresponding values in HEPES buffer are obtained (4.8 ± 1.6)×10^−3^ s^-1^, 0.48 ± 0.04, and 56.3 ± 1.8 s. These differences are due to the presence of different salts and other chemicals in the two buffers. The experimental data on ln*k*_r_ vs. 1/(*σ*_e_ + *B*) is presented in Fig 11, and the data points have been fitted using Eq (8). The best-fit values of Γ are obtained as Γ = 10.4 ± 0.9 pN for PIPES buffer and Γ = 6.8 ± 1.3 pN for HEPES buffer. Hence, the Γ for DOPG/DOPC (40/60)-GUVs in PIPES buffer is 1.5 times larger than that of HEPES buffer. The value of Γ for DOPG/DOPC(40/60)-GUVs in the PIPES buffer is very similar to that obtained in the literature [31].

## 4. Discussion

We examined the electroporation of DOPG/DOPC/GrA (40/60/0.01)-GUVs at different negative membrane potentials (*φ*_m_) while keeping electric tension (*σ*_e_) constant (Figs 3 and 4). We also investigated the electroporation of DOPG/DOPC/GrA (40/60/0.01)-GUVs at a fixed *φ*_m_ by varying *σ*_e_ (Figs 5 and 6). In both cases, we observed an increase in both rate constant of rupture (*k*_r_) and probability of rupture (*P*_rup_) with increasing *φ*_m_. Moreover, the time required for GUV rupture decreased as *φ*_m_ increased. The application of *σ*_e_ also resulted in an enhancement of electroporation kinetics. The estimated pore edge tension (Γ) is almost similar under various *φ*_m_ (Fig 8). Basically, Γ is an intrinsic membrane property originating from the physicochemical properties and the amphiphilic nature of lipid molecules in the membranes [27]. Since, the concentration of ions (whether K^+^ or a combination of K^+^ and TEAC+) in the inside and outside of GUVs remain the same for different *φ*_m_, Γ is expected to be the same for different *φ*_m_, which agrees with our estimation of Γ.

To elucidate the kinetics of electroporation in the presence of *φ*_m_, it is necessary to consider how the membrane potential induces lateral stretching in the membranes. One model predicts that the application of a potential can led to thinning of the membranes, resulting in changes in the membrane thickness (*h*) [47]. The thinning of the membrane in response to *φ*_m_ can be attributed to changes in the capacitance of the bilayer membrane. The change in capacitance can be expressed as Δ*C* = *C*_m_ (*X*_T_ + 2*X*_E_), where *C*_m_ represents the capacitance, and *X*_T_ and *X*_E_ are the fractional increases in area due to bilayer formation and elastic stress, respectively. In the case of an incompressible membrane, a decrease in membrane thickness occurs in proportion to the increase in membrane area caused by elastic stretching. The parameter *X*_E_, which represents the fractional increase in area due to elastic stress, is inversely proportional to the membrane elasticity. Therefore, a more elastic membrane will exhibit a smaller value of *X*_E_. If a change in *φ*_m_ leads to an overall decrease in membrane thickness (*h*) before rupture, it is expected that the rupture will be directly preceded by a change in the *C*_m_. This change in capacitance indicates a change in the effective area of the membrane, reflecting the thinning process. It is worth noting that the membrane volume remains essentially unchanged during this process since the volume compressibility of the membrane material is nearly constant. Therefore, the decrease in thickness is accompanied by an increase in area, while the overall volume of the membrane remains relatively constant [48,49]. Based on these researches, we have considered the additive nature of tension, such as the total membrane tension *σ*_t_ = *σ*_e_ + *σ*_φ_, where, *σ*_φ_ is induced due to the presence of *φ*_m_. The value of *σ*_φ_ depends on the magnitude of the *φ*_m_. Specifically, as the membrane potential increases, the corresponding tension induced by the membrane potential, *σ*_φ_, becomes larger. In the absence of *φ*_m_, *σ*_t_ = *σ*_e_ as *σ*_φ_ = 0.

The additive nature of tensions has been considered in several investigations, as discussed in the following studies. It has been well-reported that the presence of osmotic pressure increases the rupture of vesicles due to both mechanical tension (*σ*_m_) [50] and electric tension (*σ*_e_) [51]. These studies have explained that osmotic pressure adds an additional tension, denoted as *σ*_os_, to the externally applied *σ*_m_ or *σ*_e_. Therefore, the total tension experienced by the membranes of GUVs during electroporation can be expressed as *σ*_t_ = *σ*_e_ +*σ*_os_. Similarly, during mechanical tension in the presence of osmotic pressure, the total tension can be expressed as *σ*_t_ = *σ*_m_ +*σ*_os_. It should be noted that the tension generated due to osmotic pressure depends on the concentration gradient between the inner and outer regions of the GUVs. In another recent investigation on magainin 2-induced pore formation in lipid vesicles, the additive nature of tensions was also observed. In this case, a ‘single GUV’ was held at the tip of a micropipette, and a magainin 2 solution was introduced in the vicinity of the GUVs through another micropipette [52–54]. The total tension experienced by the membrane in this scenario can be expressed as *σ*_t_ = *σ*_mag_ + *σ*_m_, where *σ*_mag_ is induced due to the surface concentration of magainin 2.

We investigated how the energy barrier of a prepore changes with the *φ*_m_. In our analysis, as for simplicity, we considered a constant value of Γ = 6.0 pN for all cases of *φ*_m_, and a fixed value of *σ*_e_ = 6 mN/m to calculate the *k*_r_. The relationship between the energy barrier and the induced tension due to applying membrane potential across the membrane was described using Eq (7), where the parameter *A* was considered 13500 s⁻^1^. The simulated data, presented in Table 1, exhibits a strong correlation with the experimental data. This supports our proposal regarding the additive nature of *σ*_φ_ with *σ*_e_, which provides a reasonable explanation for the observed increasing trend in electroporation events (e.g., the rate constant of rupture and probability of rupture) of GUVs in the presence of *φ*_m_ (as shown in Figs 4 and 6).

**Table 1:** A comparison of rate constant between estimated and experimental data for various membrane potentials.

Membrane potential *φ*_m_ (mV)	Membrane tension due to *φ*_m_ *σ*_φ_ (mN/m)	Rate constant *k*_r_ (s^−1^) [Using Eq 7]	Rate constant *k*_r_ (s^−1^) [Expt. of Fig 8B]
0	0	7.5×10^−3^	(7.5 ± 1.6) ×10^−3^
−30	0.210	10.9×10^−3^	(10.9 ± 2.3) ×10^−3^
−60	0.465	16.9×10^−3^	(16.9 ± 2.6) ×10^−3^
−90	0.943	35.5×10^−3^	(35.6 ± 5.5) ×10^−3^

**Fig 12:**
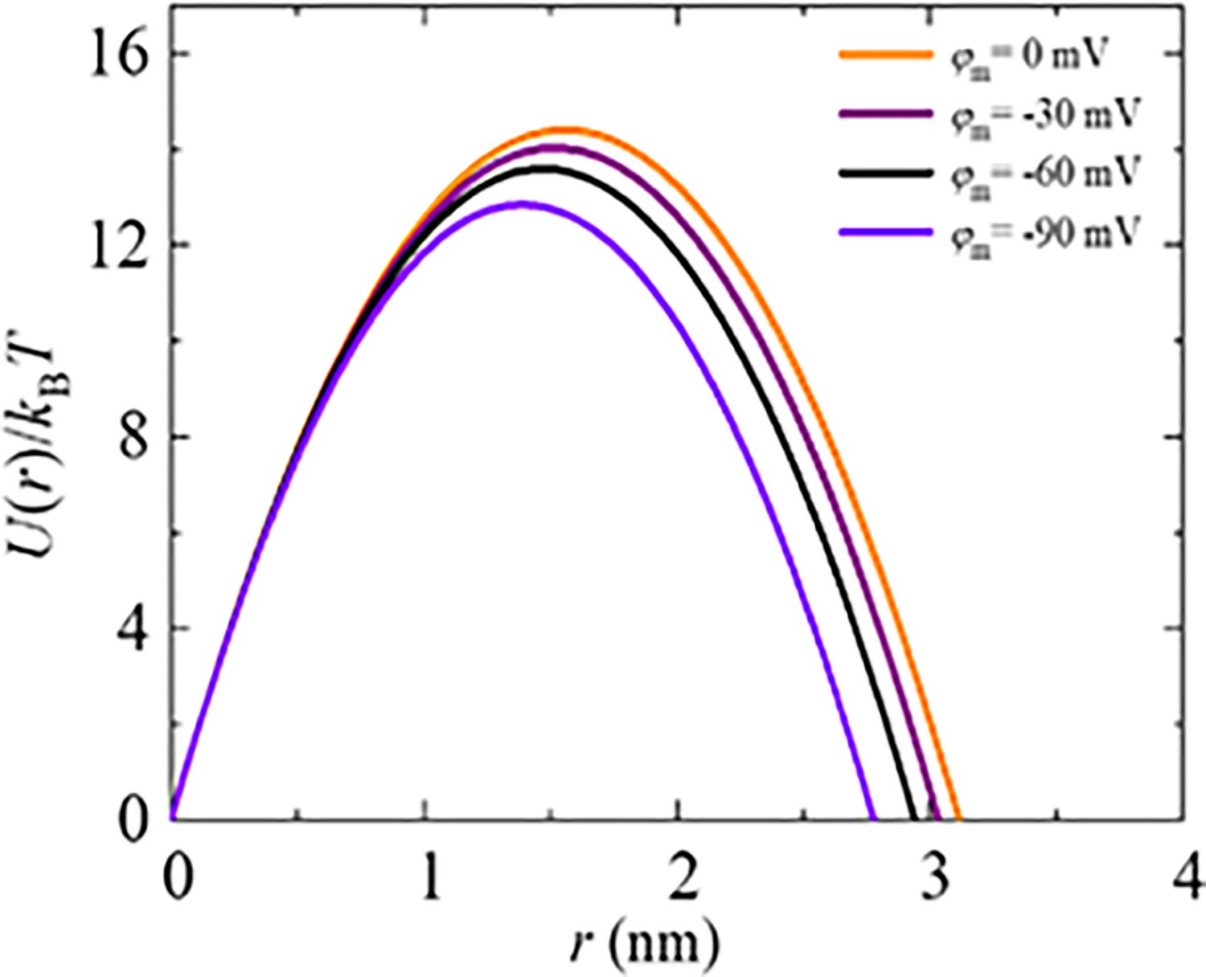
The prepore energy profile in the presence of various membrane potentials. The energy barrier is calculated using Eq (5).

The energy barrier of a prepore decreases with an increase of negative membrane potential, as depicted in Fig 12. The calculated barrier energy ranged from 14.41 to 12.84 *k*_B_*T* as *φ*_m_ increased from 0 to −90 mV. Notably, these barrier energy values closely align with those obtained in electroporation and micropipette aspiration techniques [13].

**Fig 13:**
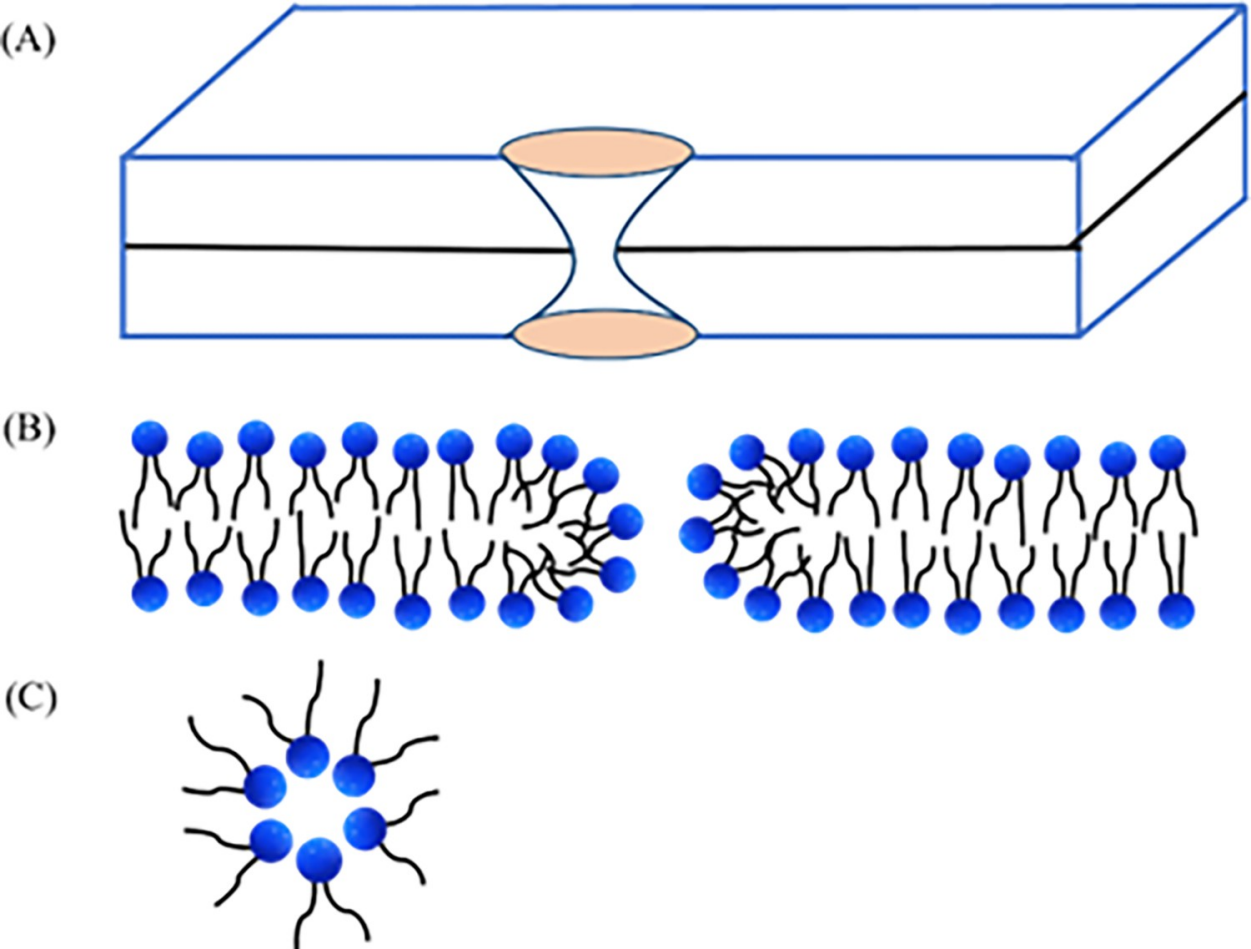
An illustration of a toroidal pore. (A) Total view, (B) side view, and (C) top view at the mid-plane of the lipid bilayer of a prepore.

The rim of a pore may be formed by the connection of the outer and inner monolayers in a toroidal fashion, as illustrated in Fig 13 [43,55]. This toroidal pore structure represents a characteristic feature of the membrane during the process of pore formation.

The primary objective of utilizing GrA was to create ion channels across the lipid bilayer. These ion channels play a pivotal role in establishing a membrane potential across the lipid membrane. It is well-documented that introducing GrA into the membrane significantly impacts the surface tension of the membranes [56]. Furthermore, GrA has a notable influence on the spontaneous curvature of the monolayer of DOPC lipid [57]. Consequently, the presence of GrA brings about changes in the mechanical properties and the spontaneous curvature of the monolayer. These alterations have a direct effect on the electroporation kinetics of DOPG/DOPC/GrA (40/60/0.01)-GUVs, leading to modified behavior in these lipid vesicles.

Numerous studies have reported that membrane potential plays a crucial role in various cellular processes, including T-cell signaling [58], antimicrobial peptide-induced pore formation, and the entry of cell penetrating peptide [32]. It has been observed that an increase in the binding constant between peptides and membranes is a key factor in enhancing poration kinetics [21]. Consequently, it has become evident from this study that the presence of *φ*_m_ facilitates increased poration and poration kinetics in lipid vesicles. This observation further supports our investigation, indicating that electroporation in GUVs is enhanced by *φ*_m_ (Figs 4 and 6).

In our study, we used KCl and TEAC salts with a combined concentration of 150 mM, similar to the previously used NaCl salt [41]. These salts are also monovalent, and their concentration was maintained at 150 mM. The lipid composition of our study remains consistent with our previously published papers, consisting of DOPG/DOPC (40/60)-GUVs [41]. Therefore, parameter *B* is considered unchanged throughout the estimation Γ. For the sake of simplicity, we did not include the term *U*_0_ in the pre-pore energy, *U*(*r*), and this omission does not have an impact on the determination of Γ.

As the value of *B* is the same as discussed in section 3.1, *U*_b_ depends on the value of *σ*_t_. With the increase of *σ*_t_, *U*_b_ decreases and thus increases the rupture kinetics according to Eq (6). As *σ*_t_ = *σ*_e_ +*σ*_φ_, where, *σ*_e_ remains constant, *σ*_t_ depends on*σ*_φ_, which is related to *φ*_m_. The *σ*_e_ dependent kinetics of DOPG/DOPC/GrA (40/60/0.01)-GUVs in the presence of a fixed *φ*_m_ shows that *σ*_e_ increases the rupture of GUVs (Fig 6), which has a similar behavior of kinetics as observed in DOPG/DOPC (40/60)-GUVs. The increase in rupture events is attributed to the decrease in the energy barrier, as described by Eq (6).

In Figs 9 and 10, we observe stochastic rupture events in individual GUVs. This stochastic behavior indicates that the initiation of pore formation in lipid membranes is a random phenomenon. The time taken to reach the critical radius of a pore (*r*_c_) varies among different GUVs. This random phenomenon has been observed in various cases [13,22,59]. For both HEPES and PIPES buffers, the rupture events, such as the rate constant and probability of rupture, increase with the applied *σ*_e_. This is because higher *σ*_e_ reduces the energy barrier required to convert a prepore to a transmembrane pore, as described in Fig 7. The values of the rate constant and probability of rupture are relatively smaller in HEPES buffer compared to PIPES buffer (Fig 10). This difference can be attributed to the variations in salts and reagents present in these buffers, which can influence the kinetics of electroporation. The results obtained for electroporation kinetics in PIPES buffer closely matched the ones reported in a previous study [38]. The value of Γ for DOPG/DOPC (40/60)-GUVs in PIPES buffer is obtained Γ = 10.4 ± 0.9 pN (Fig 11), which is well supported to the reported data such as Γ = 10.7 ± 0.4 pN [31] and 11.5 pN [60].

The membrane potential of up to −90mV is created by subjecting the GUVs to an asymmetric distribution of K^+^ in the buffer and to the addition of GrA in the bilayer. The method is in principle valid and this procedure has been employed before in the literature as we mentioned in section 2.3. It’s important to ensure that everything is in place to effectively apply the Nernst equation and create the membrane potential. However, we couldn’t conduct this specific investigation due to the required setup being unavailable. The present study involved various types of experiments, and the results were consistent with the previously reported findings, confirming the reliability of our research.

## 5. Conclusions

The electroporation of cell-mimetic lipid vesicles was studied under different membrane potentials, revealing interesting findings. It was observed that both the electroporation kinetics and the probability of rupture increased as the negative membrane potential was increased. Interestingly, the estimated pore edge tension remained relatively consistent across all the different membrane potentials tested. The addition of lateral tension due to applied electric field and the lateral tension due to membrane potential influences to decrease the barrier energy of the prepore, leading to increase the electroporation kinetics. This study presents a significant advancement in the field of electroporation technology, which holds great promise for various applications such as tumor and cancer cell ablation. Notably, this is the first reported investigation exploring the effects of membrane potential on electroporation. This study contributes to the expanding knowledge and applications of electroporation, offering new avenues for its utilization in the biomedical field.

## Supporting information

S1 File Suppl Infor_El_Pot_02.09.2023. (PDF)
