# Supplementary material for: Effects of membrane potentials on the electroporation of giant unilamellar vesicles
Source: PLoS One. 2023 Sep 12;18(9):e0291496. doi: 10.1371/journal.pone.0291496 (PMC10497157; doi:10.1371/journal.pone.0291496)
Supplement: S1 File — (PDF) [file pone.0291496.s001.pdf]

Supplementary Information (SI)

Effects of membrane potentials on the electroporation of giant unilamellar vesicles

Md. Abdul Wadud, Mohammad Abu Sayem Karal, Md. Moniruzzaman, and Md. Mamun Or Rashid

Table SI 1 Rate constant of rupture, probability of rupture, and the average time of intact DOPG/DOPC/GrA (40/60/0.01)-GUVs at σ_e = 6 mN/m in the presence of various φ_m

No.	Membrane potential, φ_m (mV)	Rate constant of rupture, k_r (s^{-1})	Probability of rupture, P_{rup}	Average time of intact GUVs, t_{intact} (s)
I	0	$(7.5 \pm 1.6) \times 10^{-3}$	0.40 ± 0.05	50.25 ± 2.1
II	–30	$(10.9 \pm 2.3) \times 10^{-3}$	0.47 ± 0.05	43.9 ± 2.0
III	–60	$(16.9 \pm 2.6) \times 10^{-3}$	0.52 ± 0.04	39.5 ± 3.1
IV	–90	$(35.6 \pm 5.5) \times 10^{-3}$	0.68 ± 0.05	28.12 ± 2.0

Table SI 2 Change in rate constant of rupture, probability of rupture, and the average time of intact DOPG/DOPC/GrA (40/60/0.01)-GUVs at σ_e = 6 mN/m in the presence of various φ_m. The data is extracted from table SI 1.

Difference	$\Delta\varphi_m$ (mV)	Δk_r (s^{-1})	ΔP_{rup}	$\lvert\Delta t_{intact}\rvert$ (s)
II–I	–30	3.4×10^{-3}	0.067	6.36
III–I	–60	9.4×10^{-3}	0.117	10.75
IV–I	–90	28.1×10^{-3}	0.283	22.13
